# Exploring racial disparities on the association between allostatic load and cancer mortality: A retrospective cohort analysis of NHANES, 1988 through 2019

**DOI:** 10.1016/j.ssmph.2022.101185

**Published:** 2022-07-31

**Authors:** Justin Xavier Moore, Sydney Elizabeth Andrzejak, Malcolm S. Bevel, Samantha R. Jones, Martha S. Tingen

**Affiliations:** a Cancer Prevention, Control, & Population Health, Medical College of Georgia, Georgia Cancer Center, Augusta University, Augusta, GA, USA; b Institute of Preventive and Public Health, Medical College of Georgia, Augusta University, Augusta, GA, USA

**Keywords:** Cancer, Life-course, Cumulative stress, Psychosocial stress, Race, Disparities, AL, Allostatic Load, BHS, Biological Health Score, BIPOC, Black, Indigenous, and People of Color, BMI, Body Mass Index, CI, Confidence Interval, CRP, C-reactive protein, CRT, Criticial Race Theory, DBP, Diastolic Blood Pressure, ICD-10, International Statistical Classification of Diseases, Injuries, and Causes of Death, NCHS, National Center for Health Statistics, NDI, National Death Index, NHANES, National Health and Nurtrition Examination Survey, NH-Black, Non-Hispanic Black, NH-White, Non-Hispanic White, PIR, Poverty Income Ratio, REGARDS, REasons for Geographic and Racial Differnces in Stroke, SBP, Systolic Blood Pressure, U.S., United States, UK, United Kingdom

## Abstract

**Background:**

Several studies suggest that chronic stress may be associated with increased risk of cancer mortality. Our study sought to determine the association between allostatic load (AL), a measure of cumulative stress, and risk of cancer death; and whether these associations varied by race/ethnicity.

**Methods:**

We performed retrospective analysis using National Health and Nutrition Examination Survey (NHANES) years 1988 through 2010 linked with the National Death Index through December 31, 2019. We fit Fine & Gray Cox proportional hazards models to estimate sub-distribution hazard ratios (SHRs) of cancer death between high and low AL status (models adjusted for age, sociodemographics, and comorbidities).

**Results:**

In fully adjusted models, high AL was associated with a 14% increased risk of cancer death (adjusted (SHR): 1.14, 95% CI: 1.04–1.26) among all participants and a 18% increased risk of cancer death (SHR:1.18, 95% CI: 1.03–1.34) among Non-Hispanic White (NH-White) adults. When further stratified by age (participants aged <40 years), high AL was associated with a 80% increased risk (SHR: 1.80, 95% CI: 1.35–2.41) among all participants; a 95% increased risk (SHR: 1.95, 95% CI: 1.22–3.12) among NH-White adults; a 2-fold (SHR: 2.06, 95% CI: 1.27–3.34) increased risk among Non-Hispanic Black (NH-Black) adults; and a 36% increased risk among Hispanic adults (SHR: 1.36, 95% CI: 0.70–2.62).

**Conclusions:**

Overall, the risk of cancer death was associated with high AL; however, when stratified among NH-Black and Hispanic adults this association was slightly attenuated.

**Impact:**

High AL is associated with increased risk of overall cancer death, and future studies should delineate the association between AL and cancer-specific mortality to better understand the causal mechanisms between cumulative stress and cancer.

## Highlights

• Allostatic load (AL) is a biologic measure for the ‘wear and tear’ of chronic stress.
• Studies suggests that racial minorities have higher allostatic load.
• There is limited knowledge on the association between allostatic and cancer, by race.
• We observed that AL may increase the risk of cancer death by 14%.
• Among those aged <40 years, high AL increased cancer death risk by more than 80%.

## 1 Introduction

### 1.1 Cancer disparities

In the United States (U.S.), cancer is the second leading cause of morbidity and mortality, responsible for an estimated 1.9 million new cases and over 608,570 deaths in 2021 alone (Siegel et al., 2021). Disparities in cancer morbidity and mortality have been observed within racial and socio-economically disadvantaged populations for decades (Carethers & Doubeni, 2020; Clegg et al., 2009; Singh et al., 2011; Singh & Jemal, 2017). Cancer disparities are mirrored by the trends observed in allostatic load (AL), an index commonly used to signify the biological wear and tear on an individual attributed to life-course stress. As suggested by *Moore* et al. (2021) (Moore et al., 2021), Hispanic and non-Hispanic Black (NH-Black) adults in the U.S. had a higher mean allostatic load compared to non-Hispanic White (NH-White) adults, irrespective of age, gender, or time period observed. In the latest time period analyzed (2015–2018), the age adjusted mean allostatic load was highest in Hispanic (3.17 male and 3.1 female) and NH-Black adults (2.86 males, 3.04 females) in comparison to NH-White adults (2.55 males, 2.44 females). Furthermore, previous literature reports a positive correlation between higher allostatic load score and increased mortality risk (Borrell et al., 2010, 2020; Castagne et al., 2018; Duru et al., 2012).

### 1.2 Understanding the role of Race(ism) on chronic stress in People of Color

Race and ethnicity are socially constructed labels, historically used to delineate and rationalize a hierarchy or dominance of one race (i.e., NH-Whites) over another (i.e., People of Color) (Flores, Serrano, Solórzano). The legend of John Henry describes the tale of an African American railroad worker during the American Reconstruction period (circa 1865–1877) who was tasked with competing against a mechanical steam drill in a famous “steel-driving” contest to build a tunnel at the mouth of the Big Bend in West Virginia. Although a markedly close competition between man and machine, John Henry emerged as the winner. Shortly thereafter competing, John Henry died from complete physical and mental exhaustion (Johnson, 1927). This folktale serves as a metaphor of the plight and lived experiences of many Black/African Americans and other marginalized racial/ethnic groups. Medical research examining racial/ethnic inequities and disparities in health outcomes often neglect the role of historical context (e.g., slavery, convict leasing, Jim Crow laws, redlining) in the manifestation of physiologic dysregulation characterized by the disproportionate and persistent hardship experienced by racial minorities. Critical Race Theory (CRT) provides a scope to examine the intersectionality of the construct of race, the history of American racism, and the subsequent forms of oppression which generate into health inequities (Flores et al.; Ford & Airhihenbuwa, 2010).

For decades, scholars of the interdisciplinary sciences (e.g., social epidemiologists, sociologists, medical anthropologists) have postulated the overwhelming toll, or physiologic tax, that psychosocial stress may inflict on Black, Indigenous, and People of Color (BIPOC) communities attributed to disproportionate treatment within their living environments. In 1994, Dr. Sherman A. James coined the term “John Henryism” in the context of minority health disparities as a synonym for prolonged, high-effort coping in response to difficult psychosocial and environmental stressors (James, 1994). The John Henryism hypothesis describes that socioeconomic deprived individuals in general, and Black people in particular, are routinely exposed to psychosocial stressors such as job insecurity, persistent financial strain, and subtle, or perhaps blatant, social insults contingent to race or social class – requiring the use of considerable amounts of energy to manage the psychological stress generated by these conditions on a daily basis (James, 1994). Similarly, in the late 1990s, McEwen and Seeman further elaborated the concept of allostatic load and “weathering” as the physiological ramification of stress, or the cumulative “wear and tear” on the body, from repeated adaptation to exogenous stressors (Felix et al., 2019; James, 1994; McEwen & Seeman, 1999; Seeman et al., 1997). Today, common examples of weathering can occur from experiencing continual racial micro-aggressions, resulting in racial battle fatigue (either consciously or sub-consciously), which in turn is expressed through various physiological symptoms (Flores et al.). In this study, we propose that repeated high-effort coping with chronic social, economic, and political adversity rooted in structural racism is an important factor in the disproportionate risk of death from cancer experienced by NH-Black adults.

### 1.3 Allostatic load and risk of cancer death

Few studies have analyzed the association between allostatic load and cancer incidence, morbidity, and mortality (Xing et al., 2020). Allostatic load may be defined or understood using varying terminology contingent upon discipline or theoretical framework. One U.S. study (Akinyemiju et al., 2020) proposed that a higher allostatic load score was associated with increased all-cause and cancer mortality among African Americans and White Americans in the REasons for Geographic and Racial Differences in Stroke (REGARDS) cohort. However, this study was limited to a sample of African American and White racial/ethnic groups, with oversampling of African Americans in the “Stroke Belt” areas of the U.S. Similarly, a prospective study in the United Kingdom (UK) (Chadeau-Hyam et al., 2020) also reported a positive correlation between increased Biological Health (BHS) score, a measurement synonymous to allostatic load, and cancer incidence. This study utilized the UK Biobank cohort which has been shown to have an over representation of White participants as well as a “healthy volunteer” selection bias (Fry et al., 2017). Moreover, the positive correlation between a higher index score and cancer mortality was likewise demonstrated in a previous NHANES analysis (Acheampong et al., 2020). While this study utilized a representative sample of the U.S. population from 1988 to 1994 via NHANES III data, it has limited knowledge of allostatic load and cancer mortality as available in more recent decades, and did not examine race-specific associations.

To date, there has been limited research on the relationship between allostatic load and cancer mortality among a current, nationally representative sample of U.S. adults. In this study, we examined the association of allostatic load and risk of cancer mortality in a representative sample of U.S. adults from 1988 to 2010; and whether these associations varied by race/ethnicity.

Examining the association of allostatic load on cancer outcomes, and whether these associations vary by race may give insight to novel approaches in mitigating cancer disparities.

## 2 Methods

### 2.1 Study design and participants

**Fig. 1:**
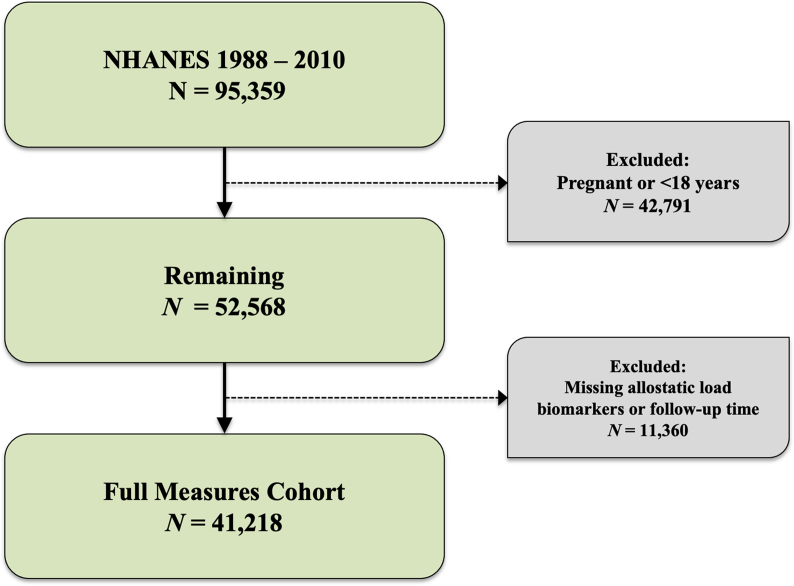
Flowchart of exclusion criteria and final study population of NHANES participants.

We performed a retrospective cohort analysis using data from National Health and Nutrition Examination Survey (NHANES), a representative sample of non-institutionalized U.S. residents, linked with the National Center for Health Statistics (NCHS) 2019 National Death Index (NDI) file. The NHANES program oversamples those aged 60 and older, Hispanic and NH-Black adults, and weighted analysis generates generalizable estimates (CDC, 2022). The NHANES weighted sample is considered to be representative of the U.S. civilian non-institutionalized population (Johnson et al., 2013). We examined the association between allostatic load and cancer using participants that completed NHANES survey from 1988 through 2010 with NDI follow-up data through December 31, 2019. NHANES includes demographic, socioeconomic, dietary, and health-related questionnaires, and includes clinical measures such as blood pressure and blood glucose, in addition to self-reported medication use for health conditions. We performed analysis among NHANES participants with data on biomarkers. We excluded participants who were missing biomarkers for allostatic load, follow up time, or were currently pregnant from this study. Moreover, participants that were missing information regarding censoring or death after NHANES linkages with the NDI were excluded due to not having information on follow up. This analysis included all NH-White, NH-Black, Hispanic, and “other” or mixed raced participants, ages 18 and older; corresponding to a total of 41,218 over the 22-year study period for the main analysis (Fig. 1). Mortality status or vital status for participants was determined through NHANES-NDI linked file. In short, NCHS investigators matched adult NHANES participants with sufficient identifying data (e.g., social security number, first and last name, sex, and date of birth) to their mortality status using information from death certificates, Social Security Administration, and Centers for Medicare and Medicaid Services. Causes of death were harmonized to International Statistical Classification of Diseases, Injuries, and Causes of Death (ICD-10) guidelines. The public use NHANES-NDI file concatenated deaths attributed to the nine leading causes of death to avoid identification of NHANES participants: these included diseases of heart, malignant neoplasms, chronic lower respiratory, accidents (unintentional injuries), cerebrovascular diseases, Alzheimer's disease, diabetes mellitus, influenza and pneumonia, nephritis, nephrotic syndrome and nephrosis.

### Ethical statement

The Institutional Review Boards considered this study exempt from review because of the use of secondary, publicly available, and de-identified data.

### 2.2 Primary exposure of interest, allostatic load

Allostatic load has been defined using varying configurations, although most incorporate biomarker measures from three different categories of physiologic functioning including cardiovascular, metabolic, and immune systems (Duong et al., 2017). While there is no consensus definition, we elected to define allostatic load using the Geronimus et al. (2006) and Moore et al. (2021) taxonomies (Geronimus et al., 2006; Moore et al., 2021). Allostatic load components included body mass index (BMI), diastolic blood pressure (DBP), glycohemoglobin (hemoglobin A1c), systolic blood pressure (SBP), total cholesterol, serum triglycerides, serum albumin, serum creatinine, and C-reactive protein (CRP). We considered sex as a biological variable according to National Institutes of Health guidelines regarding human subjects research (Arnegard et al., 2020, Lee, 2018). To determine the high-risk thresholds for each allostatic load component, we examined the gender-specific distributions of each component among the entire study sample with complete biomarker data. High-risk thresholds were determined by either being above the 75^th^ percentile for BMI, CRP, DBP, glycated hemoglobin, SBP, total cholesterol, serum triglycerides, and serum creatinine (Akinyemiju et al., 2020; Frei et al., 2015); or below the 25^th^ percentile for serum albumin. Therefore, each NHANES participant was scored as either 1 (high-risk) or 0 (low-risk) based on gender-specific cutoffs for each component (Supplemental Table 1). We calculated total allostatic load score by summing the individual components, and this score ranged from 0 to 9. We further categorized participants with allostatic load score greater or equal to 3 as having high allostatic load (Duong et al., 2017; Mays et al., 2018).

### 2.3 Primary outcome of interest, cancer death

Our primary outcome of interest was time to cancer-related death. Follow-up data for this analysis was available through December 31, 2019 based on NDI-NHANES publicly available linkages. The primary determination of mortality for eligible NHANES participants is based upon matching survey records to the NDI, although additional sources are also incorporated. These sources include the Social Security Administration, the Centers for Medicare and Medicaid Services, data collection, NCHS’ follow-up surveys, and ascertainment of death certificates. If a mortality source other than NDI was available, the participant was considered deceased. Variables indicating which source, or sources, were used to determine vital status are included in the 2019 Linked Mortality File Data Dictionary (National Center for Health Statistics).

### 2.4 Sociodemographic characteristics

Socio-demographic characteristics included in this study are age, race/ethnicity (non-Hispanic White, non-Hispanic Black, Hispanic, and other mixed race), education, and poverty to income ratio (PIR) (adjusted for inflation), and time period survey participant interviewed (1988–1991; 1991–1994; 1999–2000; 2001–2002; 2003–2004; 2005–2006; 2007–2008; 2009–2010). The NHANES education variable was categorized into: 1) less than high school education; 2) high school graduate/GED/or equivalent; 3) some college; 4) college graduate or above; and 5) unknown/refused to answer. Poverty income ratio (PIR) was calculated as the ratio of total family income to poverty threshold values (in dollars). Persons who reported having had no income were assigned a zero value for PIR. PIR values less than 1 are considered below the official poverty line, whereas PIR values greater than 1 are above the poverty level (Shargorodsky et al., 2010).

### 2.5 Health behaviors and comorbidities

We evaluated health behaviors that may influence allostatic load score in analysis, including self-reported smoking status, self-reported response to a physician-diagnosed history of cancer, as well as self-reported congestive heart failure and heart attack. Participants that had not smoked 100 cigarettes in their lifetime were categorized as never smokers, while participants with at least 100 cigarettes smoked in their lifetime but reported no current smoking use were categorized as past smokers. Participants that had smoked at least 100 lifetime cigarettes and reported current smoking use were categorized as current smokers (Bondy et al., 2009).

### 2.6 Statistical analysis

**Fig. 2:**
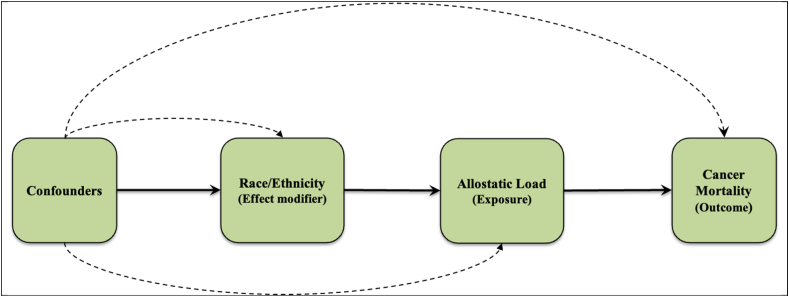
Causal diagram of the study investigation, examining the effect modification of race/ethnicity on the association between allostatic load and cancer mortality.

We performed analyses for descriptive statistics (i.e., relative frequencies and proportions for categorical variables, and means and standard errors for continuous variables) using NHANES generated sampling statistical strata, clusters, and weights as designated and described in detail within the NHANES methodology handbook (CDC, 2022). NHANES only measures biomarkers among a random sample of participants each survey period, and in turn created subsample weights to account for the probability of being selected into the subsample component, and additional non-response bias. Categorical variables were presented as weighted percentages and continuous variables as mean and associated 95% confidence intervals (CIs). We compared characteristics (i.e., descriptive statistics) by allostatic load status using Rao-Scott Chi-Square tests for categorical variables and weighted Wald F-tests for continuous variables. For time-to-event analyses, we treated our analytic cohort as a simple random sample and conducted un-weighted survival analyses. We estimated the mean survival times using the product-limit method of the Kaplan-Meier survival estimator. We examined the survival function of cancer mortality by allostatic load status overall, and then stratified by race/ethnicity using the Kaplan-Meier method. We assessed proportionality assumption for our primary exposure variable (allostatic load) by examining the proportion of 1000 simulations that contain a maximum cumulative martingale residuals larger than the observed maximum cumulative residuals using the SAS procedure ‘supremum test’. None of the levels of our exposure had p values that were statistically significant (p value < 0.05), and therefore none of our residuals were larger than expected and we did not reject proportional hazards assumptions (Grambsch & Therneau, 1994; Li et al., 2015). To estimate the relative rates of cancer death between high allostatic load and low allostatic load participants, we fit a series of Fine & Gray Cox proportional hazard models (Fine & Gray, 1999) to examine all-cause mortality as a potential competing risk for cancer deaths, and presented results from our competing risks analysis as sub-distribution hazard ratios (SHR) and associated 95% CIs. For time-to-event analysis, participants contributed to follow-up time starting from their baseline interview, and participants were censored at the time of their event, death, or end of follow-up (December 31, 2019). We sequentially adjusted our models for 1) age, 2) time period and sociodemographics (sex, race, PIR, time period, and education), and 3) health factors (smoking status, ever cancer, ever congestive heart failure, and ever heart attack). A priori we decided to examine race as an effect modifier, and thus we stratified analysis examining the association between cancer deaths by race (Fig. 2). Confounders were selected based on factors available within NHANES, biologic rationale, and bivariate analysis. We examined the multiplicative interactions of allostatic load and race/ethnicity by introducing an interaction term within our model and present the corresponding p-value for this association. Lastly, because cancer risks and allostatic load both increase with age, we performed age stratified analyses and present the effects of race and allostatic load on risk of cancer death. Age groups were categorized as less than 40 years, 40–59 years, and 60 years and older. These groups were selected due to many cancer screening guidelines and recommendations starting/initiating at age 40, and the benefits associated with cancer screening reducing by age 60+ (American Cancer Society Guidelines for, 2022). We considered p-values ≤ 0.05 statistically significant. All statistical analyses were performed using SAS (version 9.4, SAS Institute, Inc., Cary, North Carolina, USA) and Stata (version 17, StataCorp, 4905 Lakeway Drive College Station, Texas 77845 USA).

## 3 Results

### 3.1 Descriptive characteristics by allostatic load

Table 1 displays demographics of NHANES participants (n = 41,218, Fig. 1) at their baseline interview with low and high allostatic load. Participants with high allostatic load were more likely to be older (mean age 53.2 years vs. 39.4 years), more likely to identify as Non-Hispanic Black (12.9% vs. 8.4%), have a lower level of education attainment (<High School 26.3% vs. 17.9%), and be in the second quartile PIR group (19.9% vs. 17.5%) when compared to participants with a low allostatic load. Participants with high allostatic load were more likely to have a higher mean BMI (30.7 kg/m^2^, 95% CI: 30.5–30.8 vs. 25.5 kg/m^2^, 95% CI: 25.4–25.6) and less likely to report being a current smoker (23.6% vs. 25.4%) when compared to participants with low allostatic load. Participants with high allostatic load were more likely to be ever diagnosed with congestive heart failure (3.9% vs. 0.8%), heart attack (5.6% vs. 1.7%), and have any history of cancer (11.3% vs. 5.7%) when compared to participants with low allostatic load.

**Table 1:** Socio-demographic characteristics, personal health, and medical conditions by high allostatic load status, National Health Examination Survey (NHANES) study period. Among 41,218^a^ participants years 1988 through 2010 and follow up through December 31, 2019.

	High Allostatic Load (N = 19,714)	Low Allostatic Load (N = 21,504)
	Presented as N (%) or Mean (95% CL) ^b^	
**Allostatic Load Total Score**^**c**^	4.1 (0.013)	1.0 (0.008)
**Sex**		
Female	10,447 (53.4)	10,242 (48.4)
Male	9,267 (46.6)	11,262 (51.6)
**Mean Age in years**	53.2 (0.262)	39.4 (0.222)
**Age Group**		
18–29	1,560 (7.8)	7,909 (31.6)
30–39	2,328 (13.8)	4,522 (24.6)
40–49	3,228 (20.9)	3,410 (20.0)
50–59	3,188 (21.1)	2,004 (11.8)
60–69	4,223 (18.1)	1,722 (6.4)
70+	5,187 (18.2)	1,937 (5.5)
**Time Period (Year)**		
1988–1991	3940 (16.5)	3432 (14.4)
1991–1994	4919 (21.4)	2910 (12.6)
1999–2000	1357 (7.8)	2398 (11.9)
2001–2002	1603 (9.9)	2529 (12.5)
2003–2004	1520 (9.3)	2269 (11.5)
2005–2006	1645 (10.8)	2314 (11.8)
2007–2008	2366 (12.3)	2667 (12.5)
2009–2010	2364 (12.0)	2985 (13.0)
**Race/Ethnicity**		
Non-Hispanic White	9,093 (72.6)	9,985 (73.7)
Non-Hispanic Black	5,082 (12.9)	4,013 (8.4)
Hispanic	4,858 (8.9)	6,541 (11.5)
Other & Mixed Race	681 (5.6)	965 (6.4)
**Education**		
< High school	7,838 (26.3)	6,472 (17.9)
High school/GED	5,388 (30.5)	6,162 (27.7)
Some college or Associates degree	3,968 (25.1)	4,859 (27.4)
College graduate	2,444 (18.0)	3,959 (26.8)
Missing	76 (0.2)	52 (0.2)
**Income Relative to Federal Poverty Line**		
1^st^ quartile (0–1.11)	4,505 (14.8)	4,900 (14.0)
2^nd^ quartile (1.11–2.08)	4,803 (19.9)	4,654 (17.5)
3^rd^ quartile (2.08–3.77)	4,520 (26.2)	4,927 (25.9)
4^th^ quartile (3.77–11.89)	4,142 (32.1)	5,298 (36.4)
Missing	1,744 (7.0)	1,725 (6.2)
**Mean BMI, kg m**^**−2**^	30.7 (0.084)	25.5(0.048)
**Current Smoker Status**	4,317 (23.6)	4,953 (25.4)
**Any Cancer History**^**e**^	2,164 (11.3)	1,137 (5.7)
**Ever Congestive Heart Failure**	1,014 (3.9)	252 (0.8)
**Ever Heart Attack**	1,309 (5.6)	458 (1.7)

^a^ Estimated using sampling weights from National Health and Nutrition Examination Survey (NHANES).

^b^ Presented as frequency (column proportion) or mean (standard error) for continuous variables.

^c^ Allostatic load total score was calculated as sum of components based on high-risk thresholds: albumin, BMI, C-reactive protein, creatinine clearance, diastolic blood pressure, glycated hemoglobin, systolic blood pressure, total cholesterol, triglycerides. Score range from 0 to 9.

^d^ Defined as self-reported response to ever being diagnosed by a doctor or health professional of any cancer or malignancy.

^e^ Defined as self-reported response to ever being diagnosed by a doctor or health professional of any cancer or malignancy.

### 3.2 Association between allostatic load and cancer death, by race/ethnicity

**Fig. 3:**
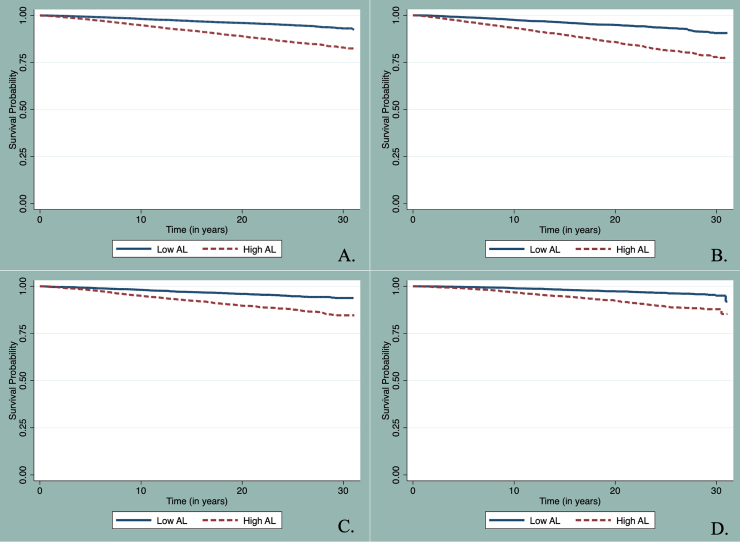
**Kaplan-Meier survival plots for time to cancer death by allostatic load. A**. Among all NHANES adults. **B**. Among NH-White adults. **C**. Among NH-Black adults. **D**. Among Hispanic adults.

In our Fine and Gray Cox Proportional Hazard models there were 2,559 deaths attributed to cancer and 8,988 deaths from other causes among our cohort. NHANES participants with high allostatic load were more likely to have death attributed to cancer when compared to those with low allostatic load (7.71% vs. 3.02%; unadjusted sub-distribution hazard ratio (SHR): 2.40, 95% CI: 2.21–2.61, Table 2) and have shorter mean survival time (27.9 years vs. 30.0 years, Log-Rank Chi-Square = 625.24, p-value < 0.01) (Fig. 3, Table 2). In fully adjusted models, all adults with high allostatic load had a 14% increased risk of cancer death (SHR): 1.14, 95% CI: 1.04–1.26) when compared to all adults with low allostatic load. When limited to NH-White adults and in fully adjusted models, those with high allostatic load had an 18% increased risk of cancer death (SHR:1.18, 95% CI: 1.03–1.34) when compared to those with low allostatic load. The associations between high allostatic load and cancer mortality were non-significant among NH-Black and Hispanic adults, but effects trended towards higher risks of cancer death (8% and 3%, for NH-Black and Hispanic, respectively).

**Table 2:** Fine & Gray method for proportional hazard models presented as Sub-Distribution Hazard ratios (SHR) and 95% confidence intervals (CI) for the association between allostatic load and cancer death accounting for competing risks of all-cause mortality, among 41,218 NHANES participants with 2,559 cancer-related deaths, and 8,988 competing deaths.

	No. Cancer Deaths (%)^a^	No.All-cause Deaths (%)^a^	Mean Survival Months (SE)^b^	Sub-Distribution Hazard Ratio (SHR) and 95% Confidence Interval (CI)			
				Unadjusted^b^	Model 1^b^	Model 2^b^	Model 3^b^
	**Risk among All Adults (*N* = 41,218)**						
**Allostatic Load**							
Low Allostatic Load	785 (3.02)	2,315 (7.85)	359.44 (0.41)	1.00 (Referent)	1.00 (Referent)	1.00 (Referent)	1.00 (Referent)
High Allostatic Load	1,774 (7.71)	6,673 (26.84)	335.45 (0.69)	2.40 (2.21–2.61)	1.28 (1.18–1.40)	1.21 (1.10–1.33)	1.14 (1.04–1.26)
	**Risk among Non-Hispanic White Adults (*N* = 19,078)**						
**Allostatic Load**							
Low Allostatic Load	453 (3.35)	1,424 (8.85)	343.12 (0.65)	1.00 (Referent)	1.00 (Referent)	1.00 (Referent)	1.00 (Referent)
High Allostatic Load	957 (8.50)	3,783 (29.48)	322.45 (1.19)	2.28 (2.04–2.55)	1.32 (1.17–1.49)	1.28 (1.13–1.45)	1.18 (1.03–1.34)
	**Risk among Non-Hispanic Black Adults (*N* = 9,095)**						
**Allostatic Load**							
Low Allostatic Load	156 (3.26)	331 (6.43)	339.69 (0.83)	1.00 (Referent)	1.00 (Referent)	1.00 (Referent)	1.00 (Referent)
High Allostatic Load	460 (6.92)	1,500 (22.88)	326.24 (1.16)	2.26 (1.88–2.71)	1.06 (0.87–1.31)	1.02 (0.83–1.26)	1.08 (0.87–1.34)
	**Risk among Hispanic Adults (*N* = 11,399)**						
**Allostatic Load**							
Low Allostatic Load	163 (1.68)	482 (3.84)	363.41 (0.59)	1.00 (Referent)	1.00 (Referent)	1.00 (Referent)	1.00 (Referent)
High Allostatic Load	323 (3.84)	1,221 (14.99)	344.67 (1.15)	2.64 (2.18–3.18)	1.14 (0.93–1.40)	1.15 (0.93–1.41)	1.03 (0.84–1.28)
**p-value for interaction**^**c**^				0.29	0.33	0.27	0.37

Model 1 is adjusted for age.

Model 2 is adjusted for age, time period, and sociodemographic factors including sex, race (only in unstratified analysis), PIR, and education.

Model 3 is adjusted for age, time period, sociodemographic factors, and health factors including current smoker status, having ever diagnosed with cancer, ever diagnosed with congestive heart failure, ever diagnosed with heart attack.

^a^ Percentages are weighted.

^b^ Models are un-weighted.

^c^ Interaction term between race/ethnicity and allostatic load on association with cancer death determined by Wald Chi-Square.

### 3.3 Association between allostatic load and cancer death, by race/ethnicity and age groups

We further examined all analyses by investigating the association between race, allostatic load, and risk of cancer death stratified by age groups using similar Fine & Gray methods for competing risks. Among those aged less than 40 years, high allostatic load was associated with up to a 2-fold increased risk of cancer death, regardless of race (Table 3; Supplemental Figs. 1–3: All adults SHR: 1.80, 95% CI: 1.35–2.41; NH-White SHR: 1.95, 95% CI: 1.22–3.12; NH-Black SHR: 2.06, 95% CI: 1.27–3.34; Hispanic SHR: 1.36, 95% CI: 0.70–2.62). When further stratified among participants aged 40–59 and 60 years and older, we observed an attenuated association between allostatic load and cancer mortality, which became less significant. Specifically, among those within the 40–59 age group, high allostatic load was associated with up to 38% increased risk of cancer death, regardless of race (All adults SHR: 1.19, 95% CI: 1.01–1.39; NH-White SHR: 1.38, 95% CI: 1.11–1.73).

**Table 3:** Age Stratified, Fine & Gray method for proportional hazard models presented as Sub-Distribution Hazard ratios (SHR) and 95% confidence intervals (CI) for the association between allostatic load and cancer death accounting for competing risks of all-cause mortality, among 41,218 NHANES participants with 2,559 cancer-related deaths, and 8,988 competing deaths.

	**No.Cancer Deaths (%)**^**a**^	**No.All-cause Deaths (%)**^**a**^		**Mean Survival Months (SE)**^**b**^	**Sub-Distribution Hazard Ratio (SHR) and 95% Confidence Interval (CI)**^**b**^
	**Age < 40 years (*N* = 16,319)**				
	**Risk among All Adults (*N* = 16,319)**				
**Allostatic Load**					
Low Allostatic Load	109 (0.92)		360 (2.48)	354.89 (0.21)	1.00 (Referent)
High Allostatic Load	80 (1.91)		302 (6.44)	356.23 (0.55)	1.80 (1.35–2.41)
	**Risk among Non-Hispanic White Adults (*N* = 5,877)**				
**Allostatic Load**					
Low Allostatic Load	47 (1.04)	113 (2.52)		354.48 (0.39)	1.00 (Referent)
High Allostatic Load	25 (2.26)	70 (6.30)		355.34 (1.18)	1.95 (1.22–3.12)
	**Risk among Non-Hispanic Black Adults (*N* = 4,209)**				
**Allostatic Load**					
Low Allostatic Load	30 (1.00)	112 (3.05)		347.68 (0.46)	1.00 (Referent)
High Allostatic Load	41 (2.11)	140 (7.98)		340.38 (0.95)	2.06 (1.27–3.34)
	**Risk among Hispanic Adults (*N* = 5,462)**				
**Allostatic Load**					
Low Allostatic Load	30 (0.58)	121 (1.93)		348.46 (0.30)	1.00 (Referent)
High Allostatic Load	13 (0.77)	82 (4.64)		320.98 (0.62)	1.36 (0.70–2.62)
	**Age 40**–**59 years (*N* = 11,830)**				
	**Risk among All Adults (*N* = 11,830)**				
**Allostatic Load**					
Low Allostatic Load	254 (3.81)	401 (5.78)		355.24 (0.98)	1.00 (Referent)
High Allostatic Load	463 (6.79)	1110 (14.66)		344.99 (0.94)	1.19 (1.01–1.39)
	**Risk among Non-Hispanic White Adults (*N* = 5,486)**				
**Allostatic Load**					
Low Allostatic Load	132 (3.77)		207 (5.78)	338.99 (1.14)	1.00 (Referent)
High Allostatic Load	219 (7.24)		446 (14.68)	330.17 (1.49)	1.38 (1.11–1.73)
	**Risk among Non-Hispanic Black Adults (*N* = 2,664)**				
**Allostatic Load**					
Low Allostatic Load	69 (6.81)		86 (9.10)	299.38 (2.22)	1.00 (Referent)
High Allostatic Load	146 (6.49)		374 (16.82)	331.43 (1.67)	0.81 (0.61–1.10)
	**Risk among Hispanic Adults (*N* = 3,177)**				
**Allostatic Load**					
Low Allostatic Load	49 (2.77)	91 (3.44)		358.96 (1.74)	1.00 (Referent)
High Allostatic Load	82 (3.17)	255 (11.39)		352.27 (1.49)	1.11 (0.77–1.59)
	**Age ≥ 60 years (N = 13,069)**				
	**Risk among All Adults (*N* = 13,069)**				
**Allostatic Load**					
Low Allostatic Load	422 (10.87)	1554 (38.77)		310.41 (2.39)	1.00 (Referent)
High Allostatic Load	1231 (12.23)	5261 (53.12)		300.99 (1.49)	1.05 (0.94–1.19)
	**Risk among Non-Hispanic White Adults (*N* = 7,715)**				
**Allostatic Load**					
Low Allostatic Load	274 (10.90)	1104 (39.79)		293.36 (2.79)	1.00 (Referent)
High Allostatic Load	713 (12.50)	3267 (54.51)		296.12 (2.20)	1.11 (0.96–1.28)
	**Risk among Non-Hispanic Black Adults (*N* = 2,222)**				
**Allostatic Load**					
Low Allostatic Load	57 (13.92)	133 (33.94)		264.75 (5.48)	1.00 (Referent)
High Allostatic Load	273 (13.55)	986 (51.46)		283.93 (2.92)	0.89 (0.66–1.21)
	**Risk among Hispanic Adults (*N* = 2,760)**				
**Allostatic Load**					
Low Allostatic Load	84 (10.77)	270 (29.60)		319.92 (4.18)	1.00 (Referent)
High Allostatic Load	228 (10.10)	884 (38.86)		303.62 (2.56)	1.07 (0.83–1.38)

Model is adjusted for time period, sociodemographic factors, and health factors including current smoker status, having ever diagnosed with cancer, ever diagnosed with congestive heart failure, ever diagnosed with heart attack.

^a^ Percentages are weighted.

^b^ Models are un-weighted.

## 4 Discussion

In a diverse, nationally representative sample of U.S. adults, we observed a 14% increased risk of cancer death among all NHANES participants with high allostatic load. Overall, when stratified by race, the association between chronic stress (i.e., allostatic load) and cancer mortality trended towards higher risk of cancer death, but was attenuated among NH-Black and Hispanic adults. When accounting for competing risk of all-cause mortality, high allostatic load was associated with 3%, 8%, and 18% increased risk of cancer death when stratified among Hispanic, NH-Black, and NH-White adults, respectively. Specifically, among those aged under 40 years, high allostatic load was associated with up to 2-fold increased risk of cancer death. There was a long-term risk of cancer death associated with cumulative stress specifically among younger adults.

### 4.1 Biological mechanism of chronic stress, inflammation, and tumorigenesis

Allostatic load is a multi-system approach to measure the biological effects of chronic stress and the over-activation of several adaptive processes that may subsequently contribute to progression of various diseases (Juster et al., 2010). Chronic stress activates the hypothalamic-pituitary-adrenal (HPA) axis and sympathetic nervous system, causing the release of corticosteroids and catecholamines respectively. Frequent exposure to these compounds have been linked to the development of cancer by DNA damage, inhibition of p53 (Feng et al., 2012), and promoting a microenvironment (Cole et al., 2015) favoring tumorigenesis (Dai et al., 2020). Chronic stress has also been shown to modulate the immune system in favor of conditions for cancer progression. In the innate immune system chronic stress and associated hormones increase pro-inflammatory cytokines (Bondar & Medzhitov, 2013). Long term pro-inflammation can influence all stages of cancer development through manipulation of tumor microenvironment (Akinyemiju et al., 2019; Coussens & Werb, 2002), genetic mutation (Lin & Karin, 2007), and epigenetic modifications (Grivennikov et al., 2010). Crucial transcription factors like STAT3 (Hodge et al., 2005) and NF-κB (Karin & Greten, 2005) in precancerous cells are activated by pro-inflammatory cytokines which can lead to genetic modifications, thereby promoting tumor survival. Furthermore, chronic stress and increased glucocorticoids (Yang et al., 2019) have a negative impact on adaptive immunity via decreased secretion of interleukin-12 (IL-12) (Curtin et al., 2009) by antigen presenting cells, and subsequent reduction in Th1 differentiation (Reiche et al., 2004; Segerstrom & Miller, 2004). Thus, chronic stress promotes favorable conditions for tumorigenesis through secretion of corticosteroids/catecholamines, increased inflammation, and immunosuppression.

Previous studies suggest that high allostatic load is associated with increased risk of all-cause mortality (Borrell et al., 2010, 2020; Castagne et al., 2018; Duru et al., 2012; Karlamangla et al., 2006), cardiovascular disease (Borrell et al., 2020; Chadeau-Hyam et al., 2020), and particularly cancer (Acheampong et al., 2020; Akinyemiju et al., 2020; Chadeau-Hyam et al., 2020; Levine & Crimmins, 2014; Robertson et al., 2017). The risk of cancer mortality in men and women has been observed between 11% and 7% respectively, among individuals with an increased BHS score, a proxy to allostatic load (Chadeau-Hyam et al., 2020). To date, there has been one other study to examine racial differences in risk of cancer mortality by allostatic load groups (Akinyemiju et al., 2020). Our observations are similar in direction of association, when compared to the prior study examining race specific association between allostatic load and cancer mortality. For instance, Akinyemiju et al. (2020), found that for every unit increase in allostatic load score there was a 6% increased risk of cancer mortality among Black participants, and 8% increased risk of cancer mortality among White participants (Akinyemiju et al., 2020); compared to 8% increased risk of cancer death in NH-Black adults in the present study. The increased association observed in our current analysis may be attributed to our larger sample size, increased number of cancer related deaths, or longer follow up time.

As defined previously, allostatic load attempts to quantify physiological stress by measuring biomarkers across cardiovascular, immune, and metabolic systems. Therefore, a higher allostatic load score can be indicative of cumulative stress over an individual's lifetime. Historically, racial and ethnic minorities face an additional complex set of adverse psychosocial challenges involving institutional and interpersonal racial discrimination (Berger & Sarnyai, 2015; Thorpe et al., 2019), which has been shown to contribute to an increased risk of many diseases (Krieger et al., 2013; Nkwata et al., 2020; Shen et al., 2019). Moore et al. (2021), reported marked disparities in the burden of high allostatic load among racial/ethnic minorities over a 30-year period, regardless of age and gender (Moore et al., 2021). Our current study did not find a significant association between elevated allostatic load and increased risk of cancer mortality among Hispanic and NH-Black adults; though there were significant associations in further age-stratified models. Several factors may explain the lack of association among Hispanic and NH-Black participants, including age, right censoring, and all-cause mortality competing risk. On average, Hispanic and NH-Black adults were younger at baseline interview when compared with NH-White counterparts (mean age 41.9 and 39.0 vs. 46.7). As a consequence, they may have had a reduced risk of cancer development and death, attributed to being 5–7 years younger. Moreover, it is plausible the attenuated effect of allostatic load on cancer mortality is explained by right censoring; the subject may have left the study before the event (cancer death occurs), and further follow-up analysis with more information regarding causes of death may elucidate more significant results. Lastly, in competing risks analysis accounting for all-cause death, both Hispanic and NH-Black participants with high allostatic load were at increased risk of cancer death. Therefore, it is feasible that while racial minorities have higher burden of allostatic load compared with their NH-White counterparts, they may experience deaths attributed to other causes at a higher rate, in turn masking the effect of allostatic load on cancer mortality when not considering competing risks.

Over our 31-year follow-up period, we observed that baseline allostatic load is associated with increased risk of cancer death. Within the age stratified Fine and Gray proportional hazard models, we were able to more effectively see the long-term effects of baseline high allostatic load (i.e., chronic stress) within the surveyed population aged less than 40 years. Since participants are surveyed at a single point in time, participants surveyed aged less than 40 years had the potential to have longer follow up time (i.e., 31 years) depending on participants respective survey year. Conversely, the opposite may be true for the surveyed participants aged 60+ years at interview. For instance, among adults aged 60+ years at interview, we are not able to determine the long-term effects of allostatic load at baseline. A plausible reason we were not able to observe an association between allostatic load and cancer death among adults aged 60+ years may be explained in part that the average life expectancy of a US adult is 78.8 years (National Vitial Statistics System, 2021). Moreover, individuals have an increased risk of cancer diagnosis starting at 40 years of age (White et al., 2014), therefore risk of cancer death among survey participants aged 60+ at interview may have survival bias or left censoring. In other words, participants aged 60+ at interview may have already had cancer and survived it (i.e., survival bias), or the event of cancer death may have happened before an individual was able to be surveyed (i.e., left censoring). For example, NH-Black adults with high allostatic load went from a 2-fold increased risk of cancer death when stratified by those aged less than 40 years, to a 19% reduced risk of cancer death when stratified by participants aged 40–59 years. In short, among those aged under 40, we are able to disentangle the life course effects of allostatic load on risk of cancer death because they have more time at risk for developing cancer. Future studies should consider longitudinal cohort designs with repeated measures of allostatic load to delineate causal associations.

### 4.2 Strengths and limitations

The results of this study should be considered in light of a few strengths and limitations. NHANES surveys a large sample of the general U.S. population, thus allowing for the analytic sample to be representative of the U.S. civilian population. This provided us the opportunity to explore the association between allostatic load and of cancer mortality among a nationally representative sample of U.S. adults, which is a limitation of smaller cohorts (Akinyemiju et al., 2020; Robertson et al., 2017). Additionally, because the NHANES survey has been collecting data for decades, our study was able to follow up survey participants for a maximum of 31 years (mean 16.9 years, median 15.7 years). While allostatic load was characterized once at baseline, it is more likely that allostatic load changes through one's life-course and may influence mortality outcomes based on the individual's lifestyle, social pressure, and coping skills. Prior studies report that allostatic load has a positive correlation with age (Moore et al., 2021), and similarly increasing age is associated with increased risk of cancer (White et al., 2014). In our study, we observed that once adjusted for age, the effect of high allostatic load on cancer attenuated, thus explaining that age is a significant predictor of cancer and is correlated with both the exposure and outcome. NHANES is a cross-sectional survey not originally intended to surveil cancer incidence and outcomes. As a result, we did not have granular information regarding cancer specificity, incidence, treatment, and progression. Given that we were unable to delineate cancer incidence, survival and differences in cancer detection, screening and treatment, it is plausible that mortality rates were variable over the study period. However, we controlled for study period that each NHANES participant was enrolled in multivariable analyses. Furthermore, NHANES collected biomarkers in a standardized manner and participants were surveyed on multiple health related behaviors and conditions, corresponding to minimal misclassification biases in our primary exposure. Future studies with more information regarding cancer patient diagnosis, treatment, and course among a large longitudinal cohort with repeated measures of allostatic load may provide more insight on the role between race, allostatic load, and cancer outcomes.

## 5 Conclusion

In this study, high allostatic load was associated with increased risk of overall cancer death, and future studies should delineate the association between allostatic load and cancer-specific mortality to better understand the causal mechanisms between cumulative stress and cancer. Allostatic load, a proxy of cumulative stress as a result of persistent environmental stimuli, is associated with increased risks of cancer mortality. Findings from our analysis continue to illuminate the deeper concerns surrounding stress and cancer-related health disparities among NH-Black and Hispanic adults. Moreover, foreign born NH-Black and Hispanic immigrants have been shown to have increasing allostatic load upon moving to the U.S. (Langellier et al., 2021). Researchers and clinicians should consider novel approaches at mitigating cancer morbidity and mortality using multi-level (i.e., community, person, inter-person, and molecular) strategies that reduce chronic stress and inflammation, such as concerted efforts towards destigmatizing mental health, and providing culturally sensitive, competent, and affordable resources in primary care facilities along the cancer care continuum.

## Ethical statement

The Institutional Review Boards considered this study exempt from review because of the use of secondary, publicly available, and de-identified data.

## CRediT authorship contribution statement

**Justin Xavier Moore:** Conceptualization, Supervision, Investigation, Visualization, Writing – original draft, Writing – review & editing, Methodology, Formal analysis, Data curation, Funding acquisition, Validation, Resources. **Sydney Elizabeth Andrzejak:** Writing – original draft, Investigation, Visualization, Formal analysis, Data curation, Validation. **Malcolm S. Bevel:** Writing – review & editing, Validation, Methodology. **Samantha R. Jones:** Writing – review & editing, Validation. **Martha S. Tingen:** Writing – review & editing, Validation.

## Declaration of competing interest

None.

## Appendix A Supplementary data

Multimedia component 1 Multimedia component 1

figs1 figs1

figs2 figs2

figs3 figs3
