# Supplementary material for: Exploring racial disparities on the association between allostatic load and cancer mortality: A retrospective cohort analysis of NHANES, 1988 through 2019
Source: SSM Popul Health. 2022 Jul 31;19:101185. doi: 10.1016/j.ssmph.2022.101185 (PMC9382324; doi:10.1016/j.ssmph.2022.101185)
Supplement: Multimedia component 1 [file mmc1.docx]

| **Supplemental Table 1: Weighted distribution of allostatic load components by gender, NHANES years 1988 through 2010, among 41,218 participants. Thresholds for each allostatic load component based on these weighted distributions.** | |
| --- | --- |
|  |  |
| **Allostatic Load Component** | **Median (Q1, Q3) ^a^** |
| **Albumin, serum (g/dL)** |  |
| Female | 4.14 (3.92, 4.35) |
| Male | 4.33 (4.12, 4.55) |
| **Body Mass Index (kg/m^2^)** |  |
| Female | 26.19 (22.60, 31.24) |
| Male | 26.89 (24.00, 30.33) |
| **C-reactive Protein (mg/dL)** |  |
| Female | 0.21 (0.13, 0.49) |
| Male | 0.20 (0.09, 0.30) |
| **Creatinine (μmol/L)** |  |
| Female | 70.71 (61.49, 79.60) |
| Male | 88.21 (79.16, 103.21) |
| **Glycohemoglobin (%)** |  |
| Female | 5.23 (4.96, 5.55) |
| Male | 5.28 (5.01, 5.56) |
| **Systolic Blood Pressure (mmHg)** |  |
| Female | 116.70 (106.76, 131.41) |
| Male | 121.54 (112.96, 132.15) |
| **Diastolic Blood Pressure (mmHg)** |  |
| Female | 69.92 (62.82, 77.70) |
| Male | 73.27 (65.93, 81.07) |
| **Total cholesterol (mg/dL)** |  |
| Female | 198.20 (172.58, 227.70) |
| Male | 196.28 (169.48, 225.11) |
| **Triglycerides, serum (mg/dL)** |  |
| Female | 103.63 (71.70, 154.97) |
| Male | 124.00 (83.03, 192.02) |
| ^a^ Estimated using sampling weights from National Health and Nutrition Examination Survey (NHANES).  Q1 = 25^th^ percentile, Q3 = 75^th^ percentile. | |
