# Supplementary figures and images for: Exploring racial disparities on the association between allostatic load and cancer mortality: A retrospective cohort analysis of NHANES, 1988 through 2019
Source: SSM Popul Health. 2022 Jul 31;19:101185. doi: 10.1016/j.ssmph.2022.101185 (PMC9382324; doi:10.1016/j.ssmph.2022.101185)

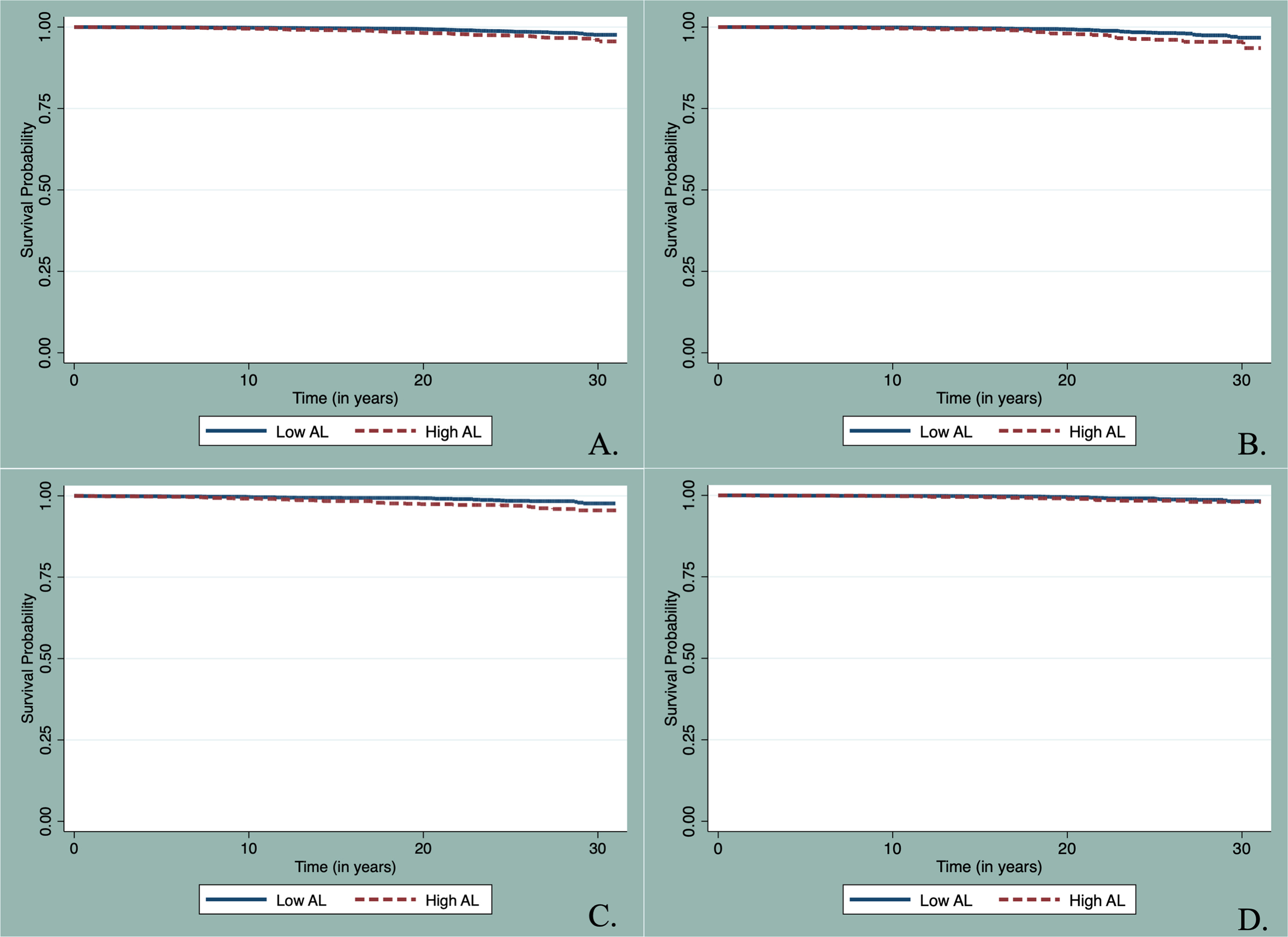

Supplement: figs1 [file mmcfigs1.jpg]

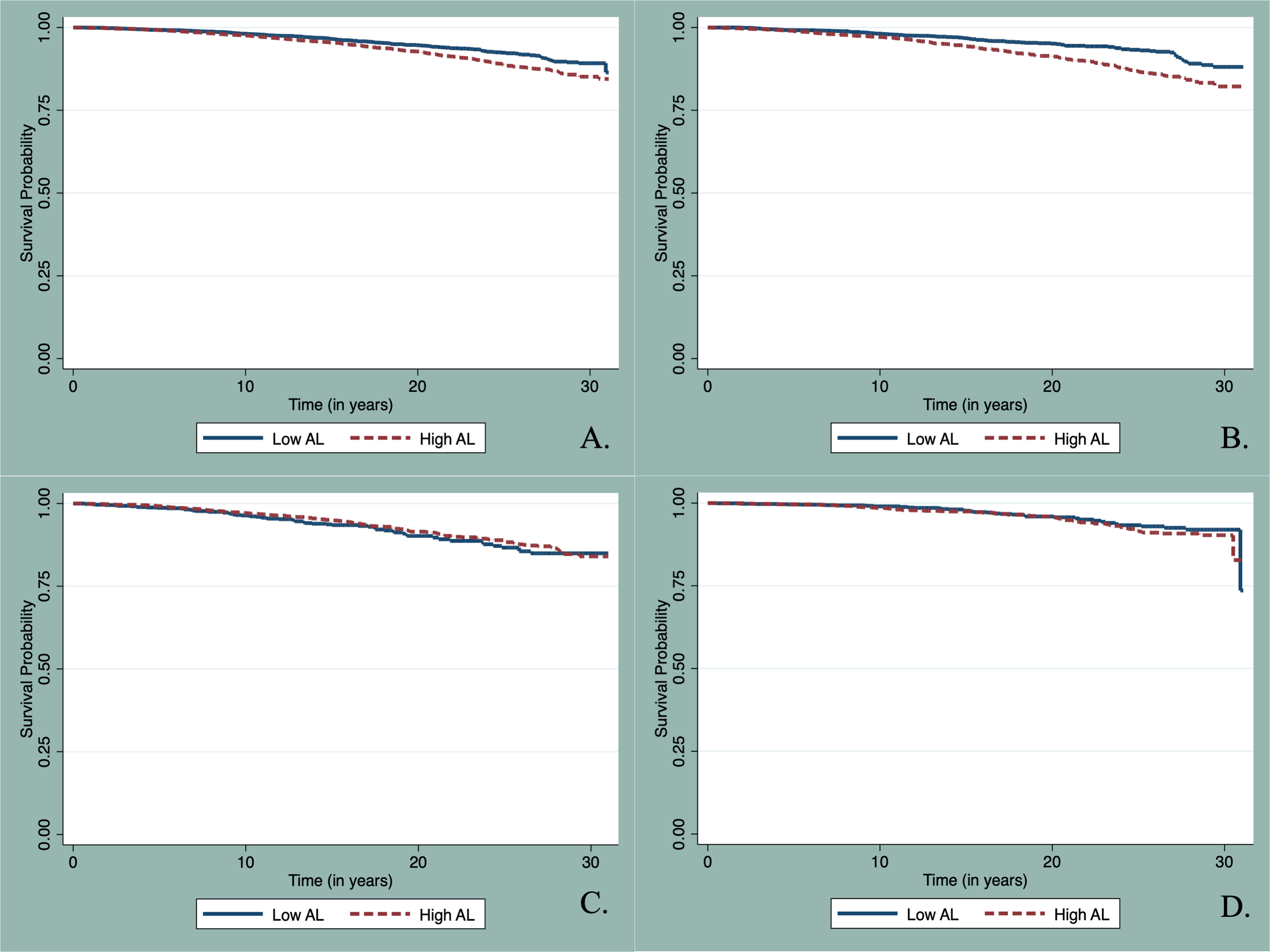

Supplement: figs2 [file mmcfigs2.jpg]

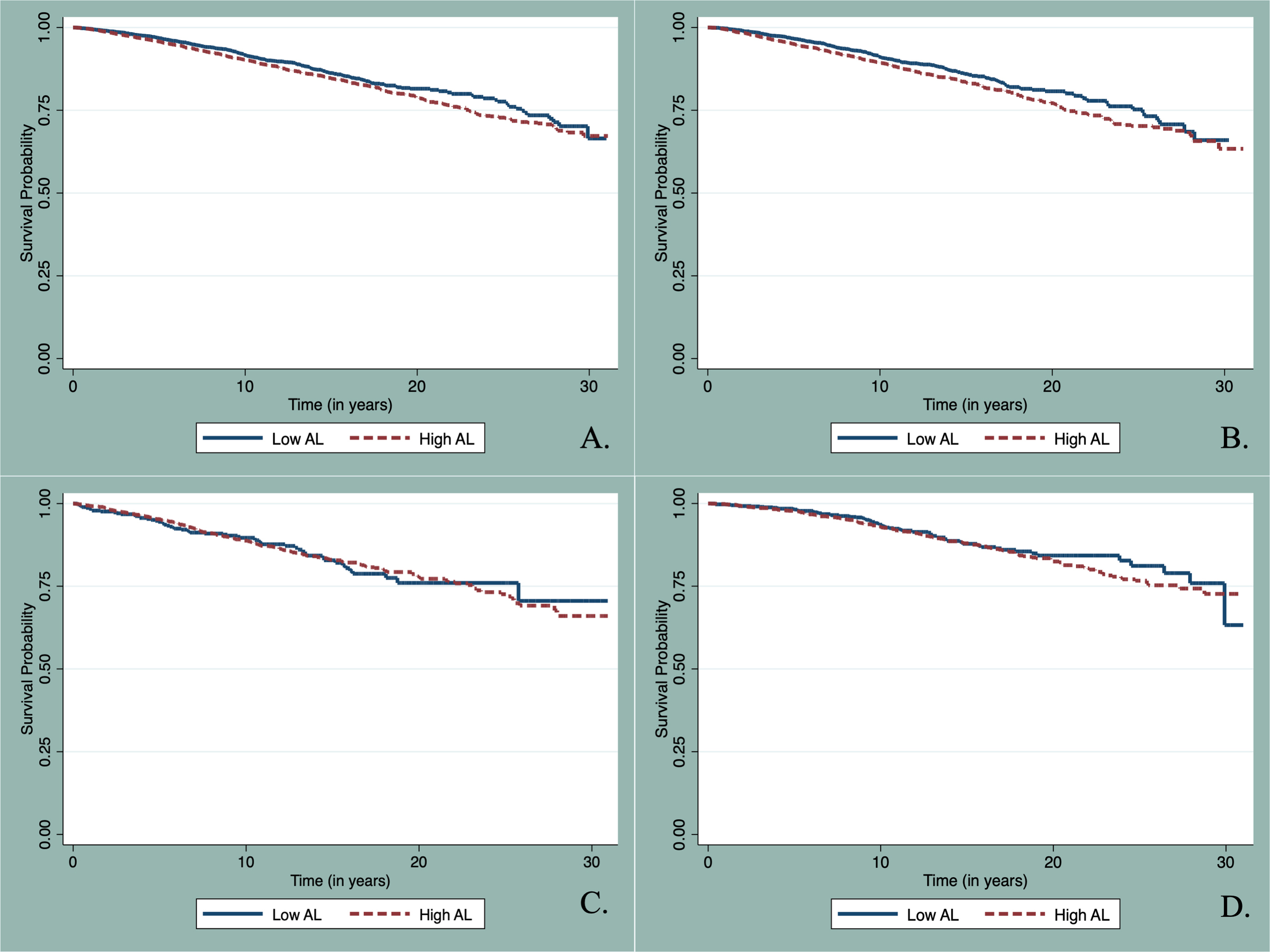

Supplement: figs3 [file mmcfigs3.jpg]
